# Timely updates to the Summary of Product Characteristics: supporting evidence-based prescribing in pregnancy

**DOI:** 10.3389/fgwh.2026.1750463

**Published:** 2026-08-28

**Authors:** Virginia Rasi, Mario Cortina-Borja, Rebecca Sconza, Claire Thorne

**Affiliations:** Population Policy and Practice Research and Teaching Department, UCL Great Ormond Street Institute of Child Health, University College London, London, United Kingdom

**Keywords:** benefit-risk assessment, breastfeeding (BF), drug safety, medicine regulation, pregnancy, real-world evidence (RWE)

## Introduction

Benefit-Risk (B-R) assessment by regulators of medicines used during pregnancy and lactation is complex. Administering medicines in pregnancy requires assessing whether the potential benefits outweigh the risks and whether treatment could detrimentally impact fetal development (1). This assessment is complicated by limited safety and efficacy data due to the systematic exclusion of pregnant individuals from registrational trials. Adequate teratogenicity evaluation also requires robust post-marketing surveillance and consideration of later outcomes such as developmental delay (2, 3). Reflecting this evidence gap, a cross-sectional analysis reported only 1.9% of all interventional trials registered on ClinicalTrials.gov specifically concern pregnancy (4).

Limited knowledge on embryofoetal toxicity and teratogenicity makes it difficult to strike a balance between the unknown risks over the potential benefits of novel therapies. Typically, in the absence of evidence, regulatory authorities adopt a precautionary approach, recommending avoidance during pregnancy and breastfeeding and focusing predominantly on fetal risk (the innocent bystander) rather than equally considering maternal and fetal benefits. This leads to fewer treatment options or increased off-label use. A recent comparison of pregnancy and lactation labelling of the US Food and Drug Agency and the European Medicines Agency (EMA) found that 90% of the evaluated drugs contained discouraging language (e.g., “should not be used”, “contraindicated”) with most recommendations based on either no or sparse human data (5). This underscores the consequences of focusing predominantly on fetal risks, without equal consideration for maternal benefits or the impact of withholding treatment. This issue is critical for women with pre-existing conditions exacerbated or complicated by pregnancy (e.g., lupus flares, poor glycaemic control), whilst pregnancy-related pharmacokinetics (PK)/pharmacodynamics (PD) changes may further affect drug effectiveness (3). Real-world safety data from observational and post-marketing studies require years to accumulate to a point where meaningful analysis is possible, and regulatory B-R re-assessments are often delayed further postponing availability of data to inform clinical decision-making. One study estimated an average of 27 years to determine pregnancy safety (6), while more recent data showed a 6-year gap between drug registration and first PK data in pregnancy for HIV medication (3).

B-R assessment of medicines used in pregnancy for chronic conditions carries additional complexity, particularly when conception occurs during treatment. Taking the example of HIV, after diagnosis, antiretroviral treatment (ART) once started should be continued for life. Many women living with HIV conceive whilst on ART and thus continue treatment throughout pregnancy, whilst those newly diagnosed initiate ART during pregnancy. ART prevents both HIV disease progression and vertical transmission (7). The case of HIV, as well as other chronic conditions where stopping medication is not possible, demonstrate the importance of considering mother and fetal exposure to both potential beneficial or detrimental medication effects throughout the pregnancy, particularly in sensitive periods such as embryogenesis (1).

## Regulatory tools and information gaps: the SmPC and EPAR

Despite the difficulties of B-R assessment, opportunities exist to enhance product information for medicines used in pregnancy and breastfeeding as new evidence emerges. The SmPC is a key regulatory document providing safety and efficacy data to support evidence-based prescribing decisions by healthcare providers (HCPs) and for patients. The SmPC is required as part of a medicine's marketing authorisation procedure and is structured into sections according to the European Commission guidance (8). SmPCs have nonetheless been criticized for inconsistent wording, lack of standardisation and suboptimal sources of key data such as food-drug interactions, or dose adjustments for specific populations (9). Although SmPCs should be regularly updated throughout a product's life-cycle, a review of 534 SmPCs available on the EMA website reported that whilst on average 5.7 years had passed since the relevant marketing authorisation had been granted with multiple updates issued (on average, more than nine), the quality of the information on pregnancy and breastfeeding, particularly placental transfer, fertility and milk excretion had not improved, with nearly 60% providing no recommendation for use and over 90% restricting use based on lack of data rather than known risks (9).

Another important source of information is the EPAR, a publicly available document summarising the scientific evaluation by the Committee for Medicinal Product for Human Use, including which of these conclusions have been integrated into the SmPCs. EPARs, available on the EMA website, include an “assessment history” section with updates documented under “Procedural steps taken and scientific information subsequent to authorization” within the assessment history section (9).

## Illustrative examples from SmPCs and EPARs for antiretroviral agents

To illustrate how pregnancy-related information is presented in regulatory documentation, we accessed a selection of publicly available SmPCs and EPARs’ “Procedural steps taken and scientific information subsequent to authorization” for EMA-approved antiretroviral agents focusing on pregnancy and breastfeeding information and how this information was updated over time (10). These examples are intended to support the discussion and should not be interpreted as a systematic review of all EMA product information. Across the selected 27 antiretroviral SmPCs (single agents and fixed dose combinations [FDC]), pregnancy-related recommendations are often unclear. For example, many provide no clear recommendation for use in pregnancy, while others advise against use through wording such as “should not be used” or “should not be initiated in pregnancy”. One-third address women of childbearing age, but this is mainly to recommend pregnancy testing and compliance with contraceptive measures during treatment. All SmPCs recommend avoidance of breastfeeding, as “a general rule” to prevent vertical transmission, yet none include a specific recommendation for the relevant agent/FDC's safe and effective use in pregnancy or lactation (10). Information on preclinical reproductive and embryofoetal toxicity, PK-PD data in pregnancy and transplacental passage is variably reported and often incomplete. For example, PK-PD data is included in just under half of the SmPCs, whilst placental transfer is addressed in only four. Terminology across the revised SmPCs is also inconsistent, with “limited data” used variably to describe either absence of data or small sample sizes (i.e., <300 observations). Harmonisation of such terms could improve consistency across regulatory documents and facilitate interpretation by HCPs.

The selected SmPCs have undergone multiple updates since marketing authorisation, mostly based on accumulating new real-world safety and effectiveness data, including pregnancy and breastfeeding use. However, even when additional pregnancy-related evidence has become available, this has not always translated into clear and timely changes in the product information. Furthermore, when updates have occurred, these have often been driven by specific safety or pharmacokinetic concerns. For example, recommendations for FDCs containing the booster cobicistat were revised following evidence of reduced exposure and an increased risk for viral rebound and vertical transmission during pregnancy (11). Taken together, these examples highlight both a strong reliance on post-marketing safety and effectiveness data and a delay to conduct PK-PD studies, resulting in knowledge gaps and potential under-dosing. Earlier inclusion of pregnant people in phase I studies could help to establish safe and effective dosing in later trimesters of pregnancy (3).

## Expanded collaborative efforts to advance pregnancy research

Regulators have recently increased their attention to pregnancy considerations in research and medicine development (12). Recent initiatives include the World Health Organization (WHO) Antiretrovirals in Pregnancy Research Toolkit developed to expand pregnancy and lactation data for HIV, viral hepatitis and sexually transmitted infections and the EMA's collaboration with the ConcePTION consortium, a European public-private initiative building on EUROmediCAT's congenital anomaly surveillance to establish a “European knowledge bank”; these should accelerate generation and communication of real-world pregnancy evidence and support timely SmPC updates related to pregnancy (12, 13). These initiatives align with the emerging ICH E21 guideline, setting out scientific and ethical principles for inclusion and retention of pregnant and breastfeeding individuals in clinical trials, marking a significant shift in medicine development.

## Case studies: contrasting regulatory responses for dolutegravir and efavirenz

However, a disconnect remains between available pregnancy data and information reflected in regulatory documents such as the SmPC. This gap is exemplified by two antiretroviral agents, dolutegravir (DTG, authorized 2014) and efavirenz (EFV, authorised 1999), both of which raised early safety signals of a potentially increased risk for neural tube defects (NTDs) with periconception exposure. Although initially based on preliminary findings the regulatory response developed differently.

For DTG, the signal emerged in 2018 from the Tsepamo Study, an observational prospective birth outcomes surveillance study, reporting an NTD prevalence of 0.94% among 426 infants with conception DTG exposure versus 0.12% among 11,300 infants exposed to other ART regimens (14). Subsequent analyses of >5,800 prospectively monitored pregnancies found no statistically significant difference in NTD risk (0.11% [95%CI 0.06, 0.19] (15), supporting revision of the initial precautionary recommendations (15). Consequently, EMA guidelines evolved progressively from the very restrictive 2018 recommendations as evidence accumulated, illustrating how responsive, data-driven reassessment can be reflected in regulatory product information (10, 15). In contrast, EFV's initial signal originated from preclinical data in cynomolgus monkeys showing fetal anomalies, including NTD, and early human case reports (16, 17). Regulators incorporated restrictive recommendations advising avoidance of EFV for childbearing, pregnant and breastfeeding individuals (10). Despite extensive subsequent evidence, including WHO-commissioned meta-analyses (18, 19), a pooled European observational study on 1,200 pregnancies (20) and over 2,000 EFV-exposed mother-infant pairs in the Tsepamo Study (15), these recommendations remain unchanged. Even with >1,000 additional first trimester exposures reported by the Antiretroviral Pregnancy Registry (APR) (21), the EFV SmPC still references the 2013 APR data and early preclinical findings (10). Notably, the compound's patent expired in 2013, coinciding with its last update (10), raising broader questions about which factors influence whether accumulated evidence is translated into regulatory updates over time. Of note, EFV was recommended in the WHO preferred first-line antiretroviral regimen up to 2018 and remains an alternative first-line agent in current WHO guidelines in specific situations.

Overall, most pregnancy restrictions appear to reflect absence of evidence rather than evidence of a safety or effectiveness issue, reflecting initial lack of pregnancy data and/or delay or inertia in updating the SmPC. The case of EFV exemplifies such inertia with over 2,000 human pregnancies still not incorporated in the SmPC. This inaction has been also described for other medicines commonly used in pregnancy such as the H1 blockerantihistamines cetirizine, loratadine and desloratadine, extensively studied including a meta-analysis on data from >200,000 pregnancies showing no teratogenic effects (22, 23). Nonetheless, their SmPC and EPAR supplements continue to advise “caution or avoidance” during pregnancy and lactation (10).

These selected examples are intended to illustrate how pregnancy-related evidence is presented in regulatory documents, rather than constituting a formal review or analysis.

## A cultural shift is needed towards timely updates and integration of pregnancy evidence

Re-evaluation mechanisms exist, yet revisions for older formulations, such as EFV, cetirizine or loratadine, are typically triggered when a potential or identified new risk arises, rather than by a more favorable B-R profile. Newer formulations show more efficient evidence-driven updates as also exemplified by raltegravir, another antiretroviral agent, EMA-authorized in 2007, which was initially not recommended in pregnancy and breastfeeding due to lack of data. Once moderate data (300–1,000 pregnancies) became available, the SmPC was revised to allow use “only if expected benefits justify potential risk” and further updated once >1,000 prospective outcomes confirmed no malformative or neonatal toxicity (10).

## Discussion

The EMA’s different approach in assessing the more recent DTG safety signal compared with the older EFV signal likely reflects a new regulatory culture recognizing the importance of both generating and making pregnancy data available as early as possible to inform decision-making by HCPs, alongside stronger commitment from originator companies to accelerate this process.

As discussed above, many calls for action have advocated for timely conduct of preclinical studies to establish reproductive and embryofetal toxicity prior to clinical trials, along with frameworks for ethical inclusion of pregnant people in registrational trials and improved capture of post-marketing data. Emerging complementary approaches, including physiologically based pharmacokinetic (PBPK) modelling, may also help bridge evidence gaps before sufficient post-marketing data become available, supporting earlier regulatory decision-making for medicines likely to be used during pregnancy. Importantly, these evolving regulatory and scientific efforts align with broader global public health priorities highlighting the potential of drug repurposing for prevention and treatment during new or reemerging epidemics (24). Ensuring updated pregnancy and lactation safety data in SmPCs can be part of preparedness activities, particularly for pathogens of high consequence for pregnancy such as Zika virus, for which rilpivirine, another antiretroviral agent, has been explored as a repurposing candidate (25).

We therefore suggest that a possible regulatory approach would be to make SmPC updates a more proactive component of regulatory life-cycle management, as part of broader efforts to improve pregnancy and breastfeeding in clinical research and to accelerate studies of new medicines in pregnancy.

Rather than relying predominantly on the emergence of new safety concerns, periodic reassessment could also be triggered when predefined bodies of evidence become available, including from PK studies in pregnancy, PBPK modelling, high-quality observational cohorts, pregnancy registries, or consistent real-world evidence supporting a more favourable B-R profile. Such an approach could facilitate more timely, transparent and consistent integration of pregnancy-related evidence into regulatory product information while maintaining existing regulatory standards. Ultimately, this could better support HCPs and pregnant people in making informed treatment decisions, particularly for chronic conditions requiring continuous therapy.
